# *Eurotium cristatum* Solid-State Fermentation of Burdock Roots: Nutritional Changes, Enhanced Antioxidant Capacity, and Its Association with Phenolic Remodeling

**DOI:** 10.3390/antiox15070811

**Published:** 2026-06-28

**Authors:** Xiaoyu Yang, Xiaoxiao Jiang, Zijun Liu, Jiawei Zhang, Jinyu Yang, Shuangzhi Zhao, Dafeng Jiang, Xiangyan Chen, Qingxin Zhou, Leilei Chen

**Affiliations:** 1 Institute of Food & Nutrition Science and Technology, Shandong Academy of Agricultural Sciences, Jinan 250100, China; 2 National Center of Technology Innovation for Comprehensive Utilization of Saline-Alkali Land, Dongying 257347, China; 3 School of Bioengineering, Qilu University of Technology (Shandong Academy of Sciences), Jinan 250353, China; 4 NHC Specialty Laboratory of Food Safety Risk Assessment and Standard Development (Food Microorganisms), Shandong Center for Disease Control and Prevention, Jinan 250014, China; 5 Chengyang Campus, Qingdao Agricultural University, Qingdao 266109, China

**Keywords:** burdock roots, *Eurotium cristatum*, fermentation, antioxidant activity, phenolic compounds

## Abstract

Solid-state fermentation of burdock roots with *Eurotium cristatum* was performed to enhance their functional properties. Fermentation induced marked compositional remodeling, resulting in a 1.37-fold increase in protein content compared to unfermented controls. Antioxidant capacities were markedly enhanced. DPPH and ABTS radical-scavenging activities both exceeded 90%, and intracellular ROS levels in *Caenorhabditis elegans* were reduced by 62.7%. Phenolic profiling via UPLC-ESI-MS/MS identified and quantified 74 phenolic compounds across samples; notably, 10 flavonoids were exclusively detected in fermented burdock roots, indicative of microbial biotransformation. Correlation analysis integrating phenolic abundance with all three antioxidant endpoints revealed 11 compounds significantly associated with enhanced bioactivity. Among these, sinapic acid, 3-hydroxyflavone, liquiritigenin, and sakuranetin exhibited positive correlations with all three antioxidant measures. Prostaglandin G/H synthase 1 (PTGS1) and PTGS2 were identified as shared antioxidant-relevant targets, with PTGS1 highlighted due to its constitutive role in prostaglandin biosynthesis. Importantly, 3-hydroxyflavone, liquiritigenin, and sakuranetin were newly emerged following fermentation, providing direct evidence that *E. cristatum* mediates the synthesis or structural modification of key flavonoids, thereby augmenting the antioxidant chemical profile and functional efficacy of burdock roots.

## 1. Introduction

Burdock (*Arctium lappa* L.) is a biennial herb belonging to the Asteraceae family and native to temperate regions of Europe and Asia. Its main edible part is the burdock root, which has both medicinal and edible uses [1]. It is rich in polysaccharides, proteins, amino acids, and phenolic compounds [2]. Caffeoylquinic acid derivatives of burdock root have been demonstrated to have protective effects against oxidative stress in neuronal cells [3]. In addition, bound polyphenols from burdock root dietary fiber have been reported to attenuate inflammatory responses by reducing intracellular ROS generation and enhancing antioxidant enzyme activities [4]. Other phenolics identified in burdock root include flavonoids (e.g., quercetin, luteolin, and their glycosides) and phenolic acids (e.g., chlorogenic acid and caffeic acid), which may also contribute to its antioxidant and anti-inflammatory properties [5]. Beyond these antioxidant-related effects, contemporary scientific studies have also confirmed its hypolipidemic and anti-diabetic activities [6,7]. These nutrients and bioactive compounds endow the burdock root with health benefits.

Beyond its use as a fresh vegetable or raw materials in traditional Chinese medicine, burdock root is mainly processed into functional teas across East and Southeast Asia [8]. Microbial fermentation is a traditional food processing strategy that enhances both the sensory attributes and nutritional bioavailability of plant-based substrates [9]. In recent years, lactic acid bacteria and other microbial cultures have been increasingly applied in the biotransformation of burdock root to alleviate its off-odor and expand its application potential in functional food development [10,11]. Nevertheless, the studies have mainly focused on the sensory evaluation and volatile profile modulation of burdock roots, with less attention paid to the changes in their nutritional components, active ingredients, and biological efficacy after fermentation.

*Eurotium cristatum*, commonly known as the “golden flower fungus” due to its characteristic yellow ascocarp morphology, is the dominant and functionally important fungus in traditional Chinese Fu brick tea [12]. As a probiotic fungus, *E. cristatum* has strong growth ability and a rich enzyme system, including cellulases, tannases, and glycosidases. In recent years, its application has expanded beyond tea to diverse plant materials such as Ginkgo biloba seed, Angelica dahurica, buckwheat, and soybean [13,14,15,16]. Through enzymatic hydrolysis and microbial metabolism, *E. cristatum* facilitates the release of bound phenolics, the deglycosylation of flavonoid glycosides into more bioavailable aglycones, and the de novo synthesis of bioactive metabolites. Thereby, fermentation by *E. cristatum* enhances the nutritional and functional properties of the plant substrates. However, there are few studies of *E. cristatum* fermentation in the processing of burdock roots.

This study systematically evaluated the impact of solid-state fermentation mediated by *E. cristatum* on the nutritional composition, in vitro and in vivo antioxidant capacity, and phenolic metabolic profile of burdock root. Through correlation analysis, key phenolic compounds responsible for the elevated antioxidant activity in the fermented burdock roots were identified. These findings not only advance the understanding of phenolic biotransformation by *E. cristatum* but also highlight the potential of targeted fermentation as a strategy to strengthen the antioxidant efficacy of plant-derived functional ingredients.

## 2. Materials and Methods

### 2.1. Materials and Chemical

Dried burdock roots were purchased from Shandong Shengchuan Food Technology Co., Ltd. (Anqiu, Shandong, China). Burdock was planted uniformly in Anqiu City by Shandong Shengchuan Food Technology Co., Ltd. The region features a temperate monsoon climate with well-defined seasonal variation, and its deep, loamy soil provides optimal growing conditions for burdock. *E. cristatum* (NCPS2018015) was isolated from fermented tea in our laboratory. The strain was stored in the CCTCC (China Center for Type Culture Collection, Wuhan University, Wuhan, China) with the deposit number CCTCC NO: M2018882. Coomassie Brilliant Blue G-250, bovine serum albumin (BSA), ninhydrin, L-glutamic acid, glucose and DNS were obtained from Solarbio Co. (Beijing, China). Phenol was obtained from Aladdin Co. (Shanghai, China), and 2′,7′-dichlorodihydrofluorescein diacetate (DCF-DA) was purchased from Sigma-Aldrich Co. (St. Louis, MO, USA). Phenolic standards were purchased from Shanghai Yuanye Biotechnology Co., Ltd. (Shanghai, China).

### 2.2. Solid-State Fermentation of Burdock Roots

*E. cristatum* was cultured on a malt extract medium plate at 28 °C for 6 days to activate the strain. Until *E. cristatum* produced abundant golden spores on the plate surface, the spores were harvested by rinsing with sterile water to obtain a spore suspension. Spore counts were determined by a hemocytometer, and the suspension was adjusted to 10^6^ spores/mL.

Dried burdock roots were washed and soaked in sterile water for 1 h. Then, the water was squeezed out to ensure that the moisture content of the burdock reached 50%. 50 g of hydrated burdock roots were sterilized at 121 °C for 1 h. After cooling to room temperature, the sterilized material was inoculated with 2.5 mL of spore suspension. The inoculated burdock roots were cultured at 28 °C for 6 days. Based on preliminary antioxidant activity assays (DPPH and ABTS) and visual monitoring of fungal growth kinetics, days 1, 3, 4, and 6 were identified as representative time points capturing the critical stages of bioactivity changes and culture development. Accordingly, sterilized burdock root samples, along with those collected on the 1st, 3rd, 4th, and 6th days of fermentation, were designated as UB, D1, D3, D4, and D6, respectively. Samples were frozen with liquid nitrogen and then transferred to a freeze dryer (SCIENTZ-30F, Ningbo Scientz Biotechnology Co., Ltd., Ningbo, China). The freeze-drying process was conducted under a vacuum pressure of <10 Pa, with a programmed temperature gradient from −50 °C to 25 °C by a total duration of 48 h. The lyophilized samples were ground and stored at −80 °C for further analyses. Triplicate preparations were made for each sample.

### 2.3. Determination of Biochemical Components

#### 2.3.1. Determination of Soluble Proteins

Soluble protein content in burdock samples was quantified using the Bradford assay [17]. Briefly, 1.0 g of lyophilized burdock powder was homogenized in 50 mL of deionized water and subjected to ultrasonic extraction for 15 min at 4 °C. Following centrifugation at 10,000× *g* for 10 min at 4 °C, the supernatant was collected. For colorimetric detection, 100 μL of supernatant was mixed with 1.0 mL of Coomassie Brilliant Blue G-250 reagent (0.1 mg/mL), vortexed thoroughly, and incubated for 5 min in the dark. Absorbance was measured at 595 nm using a spectrophotometer (NanoPhotometer C40, IMPLEN GmbH, Munich, Germany). Protein concentration was calculated based on a standard curve generated with bovine serum albumin (BSA) solutions. The soluble protein content was expressed as milligrams of protein per gram of dry weight (mg/g).

#### 2.3.2. Determination of Free Amino Acids

Free amino acid content in burdock samples was determined using the ninhydrin assay [18]. Briefly, 1.0 g of burdock powder was suspended in 50 mL of deionized water, boiled for 15 min under reflux, and cooled to room temperature. Centrifugation was performed at 10,000× *g* for 10 min at 4 °C; the supernatant was then harvested as the aqueous extract. For quantification, 200 μL of extract was mixed with 1.0 mL of ninhydrin reagent (containing cadmium chloride as catalyst), heated at 100 °C for 15 min, and then cooled on ice. Absorbance was measured at 570 nm using a spectrophotometer. A standard curve was constructed using L-glutamic acid solutions, and free amino acid content was calculated based on the L-glutamic acid standard curve. The free amino acid content was expressed as milligrams of L-glutamic acid equivalents per gram of dry weight (mg/g).

#### 2.3.3. Determination of Total Sugars

The total sugar contents in the burdock samples were quantified using the phenol–sulfuric acid assay [19]. Briefly, 1.0 g of burdock root samples were extracted with 25 mL of distilled water under reflux at boiling temperature for 30 min. An aliquot of the clarified extract was reacted with 1.0 mL of 6% (*w*/*v*) aqueous phenol solution, and then 5.0 mL of 98% (*v*/*v*) sulfuric acid was rapidly introduced. The mixture was vortexed thoroughly, incubated at room temperature for 20 min, and subsequently cooled to room temperature. Absorbance was measured spectrophotometrically at 490 nm against a reagent blank. The total sugar content was calculated based on the glucose standard curve. The total sugar content was expressed as milligrams of glucose equivalents per gram of dry weight (mg/g).

#### 2.3.4. Determination of Reducing Sugars

The content of reducing sugar in burdock samples was determined by the DNS method [20]. The aqueous extract of burdock powder extracted by boiling water bath made in 2.3.3 was mixed with DNS reagent, and then the reaction was conducted in a boiling water bath for 5 min. The absorbance of the resulting mixture was read at 540 nm. The content of reducing sugar was calculated according to the glucose standard curve and expressed as milligrams of glucose equivalents per gram of dry weight (mg/g).

### 2.4. Measurement of DPPH and ABTS Free Radical Scavenging Capacity

DPPH and ABTS scavenging capacity were measured using the respective Assay Kit from Solarbio Science & Technology Co., Ltd. (Beijing, China).

### 2.5. Measurement of the ROS Scavenging Activity in Living C. elegans

The reactive oxygen species (ROS) scavenging activity of burdock root was determined in live *Caenorhabditis elegans* using the 2′,7′-dichlorodihydrofluorescein diacetate (DCF-DA) molecular probe [21]. Burdock root powder was extracted with methanol, dried, and dissolved in DMSO (1 mg/mL); the extract was diluted 200-fold with OP50. Synchronized L4-stage *C. elegans* N2 worms were transferred to these plates (80 worms/plate, two plates/group) and maintained for 4 days with daily transfer. For ROS detection, treated worms were washed, and 15 worms per well (six replicates) were loaded into a black 96-well plate containing 50 μL M9 buffer, followed by the addition of 50 μL DCFH-DA (50 μmol/L). The microplate was incubated at 20 °C for 4 h on an orbital shaker and detected with a microplate reader. Immediately prior to measurement, plates were shaken for 30 s to ensure uniform nematode distribution, and fluorescence was recorded at 485 nm (excitation) and 535 nm (emission). Values were normalized to the DMSO-treated control (set to 100%) and reported as mean ± SD from three independent biological experiments.

For imaging, ten L4/young adult worms were incubated with 10 μmol/L DCFH-DA at 37 °C for 2 h, washed, paralyzed with 25 mmol/L levamisole, mounted on agarose pads, sealed, and imaged by fluorescence microscopy with identical exposure settings to visually confirm the changes in ROS levels.

### 2.6. Analysis of Phenol Compounds in Burdock Roots by UPLC-ESI- MS/MS

Phenolic compounds in burdock root samples were qualitatively and quantitatively analyzed by UPLC-ESI-MS/MS. Analyses were performed on an ExionLC AC UPLC system (AB SCIEX, Framingham, MA, USA) interfaced with a QTRAP 6500+ hybrid triple quadrupole/linear ion trap mass spectrometer (AB SCIEX). Chromatographic separation was achieved on a Waters ACQUITY UPLC HSS T3 column (2.1 × 100 mm, 1.8 μm) maintained at 40 °C. A 5 μL aliquot of each sample extract was injected, and separation was carried out at a flow rate of 0.35 mL/min using a binary gradient mobile phase consisting of solvent A (0.1% *v*/*v* formic acid in ultrapure water) and solvent B (acetonitrile, LC–MS grade). The gradient program was as follows: 0 min A/B (95:5, *v/v*, 0.8 min A/B (95:5, *v/v*), 3 min A/B (75:25, *v/v*), 12 min A/B (56.2:43.8, *v/v*), 13 min A/B (1:99, *v/v*), 14.4 min A/B (1:99, *v/v*), 14.41 min A/B (95:5, *v/v*), 15 min A/B (95:5, *v/v*). Mass spectrometric detection was conducted in both positive- and negative-ion electrospray ionization (ESI) modes under optimized source parameters. Compound identification was based on retention time, accurate mass ([M + H]^+^ or [M − H]^−^), and diagnostic MS/MS fragmentation patterns, while absolute quantification was performed using the selected reaction monitoring (SRM) mode of the triple quadrupole mass spectrometer.

Absolute quantification of phenolic compounds was performed using external standard calibration. Mixed standard solutions were prepared at a series of concentrations and analyzed under the same UPLC-ESI-MS/MS conditions. Standard curves were constructed by plotting the peak area of each analyte against its nominal concentration, and linearity was assessed by the coefficient of determination (r^2^), which exceeded 0.99 for all analytes. To monitor extraction efficiency and instrument stability, succinic acid-2,2,3,3-d4 and salicylic acid-d4 were spiked as process internal standards, and L-2-chlorophenylalanine was added as an injection internal standard. Quality control (QC) samples, prepared by pooling equal aliquots of all sample extracts, were injected periodically. The relative standard deviation (RSD) of the peak areas for all target compounds in the QC samples was below 20%, confirming excellent analytical precision and system stability. Detailed method validation parameters for each phenolic compound are provided in Table S1. All concentration values are presented as absolute concentrations (μg/g dry weight).

### 2.7. Network Pharmacology Analysis and Antioxidant Targets Screening

Targets of the four phenolic compounds were retrieved from the TCMSP database (https://www.tcmsp-e.com/, accessed on 21 April 2026) using default criteria (OB ≥ 30%, DL ≥ 0.18). All target identifiers were standardized to official gene symbols via UniProt (https://www.uniprot.org/, accessed on 21 April 2026, species: Homo sapiens), and non-human or unannotated entries were removed.

To prioritize targets associated with antioxidant activity, a Python-based keyword matching approach was applied. Standardized target annotations were screened against a curated list of antioxidant-related Gene Ontology terms and pathway keywords, including “oxidative stress response”, “Nrf2 antioxidant pathway”, “ROS metabolic regulation”, and “redox homeostasis”. Only targets matching at least one keyword were retained.

The filtered targets were analyzed using the STRING database (v11.5) with a medium confidence interaction score > 0.4. The resulting protein–protein interaction (PPI) network was visualized in Cytoscape 3.9.1 and topologically analyzed using Network Analyzer plug-in Cytoscape 3.9.1. Hub targets were ranked by connectivity degree; the node with the highest degree was identified as the central candidate.

### 2.8. Statistical Analysis

All experiments and fermentative processes were performed independently in triplicate. Data were analyzed using Graphpad Prism 8.0, Origin 2025, and Excel 2019. One-way analysis of variance (ANOVA) and Student’s *t*-test were employed to assess statistically significant differences among group means. A significance threshold of *p* < 0.05 was adopted for all tests. Data visualization was performed using Origin 2021.

## 3. Results and Discussion

### 3.1. Effects of E. cristatum Fermentation Bioconversion on the Content of Nutritional Components in Burdock Root

**Figure 1:**
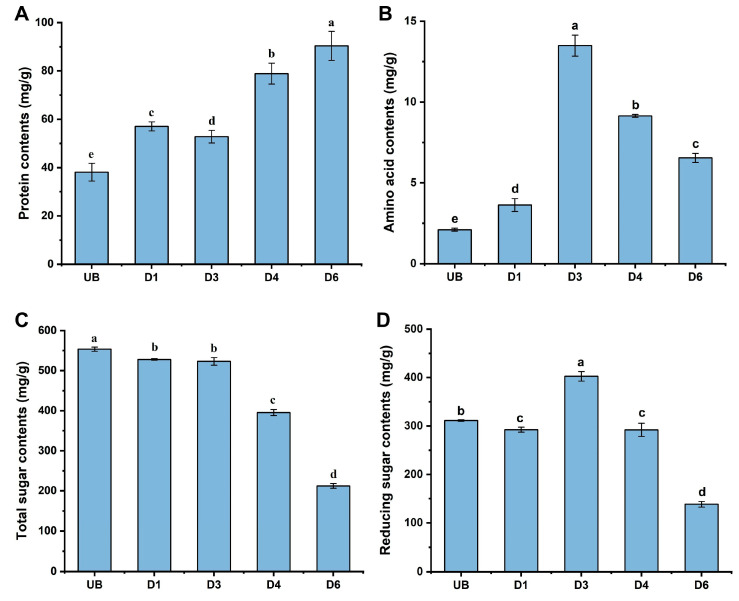
Changes in the contents of (**A**) soluble proteins, (**B**) free amino acids, (**C**) total sugars, and (**D**) reducing sugars of burdock roots during fermentation. UB: unfermented burdock roots; D1: burdock roots fermented for 1 day; D3: burdock roots fermented for 3 days; D4: burdock roots fermented for 4 days; D6: burdock roots fermented for 6 days. Different lowercase letters (a–e) indicate statistically significant differences (*p* < 0.05).

To investigate the impact of *E. cristatum* fermentation on the nutritional components of burdock roots, we analyzed the changes in soluble proteins, free amino acids, total sugars, and reducing sugar contents during the fermentation process. As shown in Figure 1A, the soluble protein content in burdock exhibited an overall upward trend with prolonged fermentation time. After 6 days of fermentation with *E. cristatum*, the soluble protein content increased from 38.13 mg/g to 90.39 mg/g, representing a 137.1% increase compared to unfermented burdock. However, a slight decrease in soluble protein content was observed on the third day of fermentation. In contrast, the free amino acid content displayed an initial increase followed by a decline (Figure 1B). On the 3rd day, the free amino acid content rose from 2.10 mg/g to 13.49 mg/g, but subsequently decreased to 6.55 mg/g after 6 days of fermentation. The observed variation in amino acid content corresponded to changes in protein levels during fermentation. The increase in soluble protein content can be attributed to two complementary processes. First, *E. cristatum* undergoes vigorous growth during the early to middle stages of fermentation, which contributes fungal biomass and associated mycoproteins to the substrate. Second, macromolecular insoluble proteins in burdock roots are progressively modified and solubilized through the action of extracellular enzymes secreted by the fungus [22]. During the early growth phase, *E. cristatum* secretes proteases to support its metabolic activity and biomass accumulation. Partial proteins were degraded into peptides and free amino acids, leading to a substantial increase in free amino acid content observed in the burdock fermentation substrate within the first three days [23]. Subsequently, a marked decline in free amino acid levels and a consistent rise in soluble protein concentrations were noted after this initial period. We speculated that the proliferation of *E. cristatum* facilitates an increase in mycoprotein, accompanied by the re-catalysis and synthesis of free amino acids into polypeptides, thereby augmenting soluble protein content and reducing free amino acid levels. A decrease in amino acid content following fermentation has also been documented during the fermentation of various other substrates, such as dried longan and Pu-erh tea [24,25]. Free amino acids may undergo degradation into volatile acids, amines, and other nitrogenous compounds, contributing further to their diminished concentration [26]. As the fermentation period progressed, the total sugar content in burdock root exhibited an overall declining trend (Figure 1C). After 6 days of fermentation with *E. cristatum*, the total sugar content decreased from 0.55 g/g to 0.21 g/g, representing a reduction of 61.8% compared to unfermented burdock. In contrast, the reducing sugar content initially increased and subsequently decreased, reaching a peak value of 0.40 g/g on the third day before declining (Figure 1D). *E. cristatum* secreted cellulase, pectinase, and other hydrolases capable of hydrolyzing cellulose, pectin, and related plant polysaccharides, thereby elevating reducing sugar concentrations during the initial fermentation stage [27]. Meanwhile, *E. cristatum* utilized sugars in burdock root as its carbon source, leading to a sustained reduction in both total and reducing sugar contents. This decline accelerated markedly during the late fermentation stage, particularly when fungal biomass reaches elevated levels [23]. Further studies measuring fungal biomass dynamics and the activities of key lignocellulolytic enzymes (e.g., cellulase, tannase, β-glucosidase) would be valuable to fully elucidate the mechanistic basis of the observed compositional changes.

### 3.2. Effect of E. cristatum Fermentation on the Antioxidant Activity of Burdock Root

**Figure 2:**
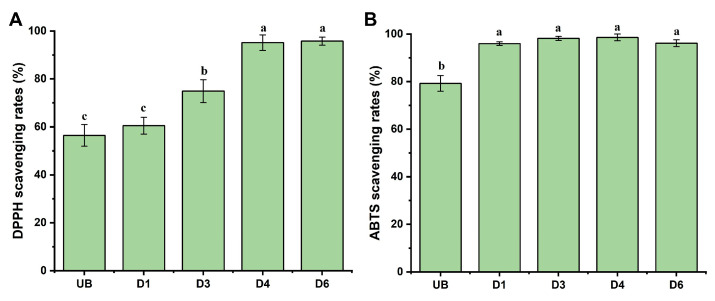
Changes in antioxidant activities in vitro of burdock roots during fermentation. (**A**) DPPH radical scavenging activity. (**B**) ABTS·radical scavenging activity. UB: unfermented burdock roots; D1: burdock roots fermented for 1 day; D3: burdock roots fermented for 3 days; D4: burdock roots fermented for 4 days; D6: burdock roots fermented for 6 days. Different lowercase letters (a–c) indicate statistically significant differences (*p* < 0.05).

Free radicals are a type of highly reactive substance with strong oxidation activity. When free radicals are in excess, they can induce oxidative reactions in the body and cause damage to tissues and cells [28]. Therefore, substances that can eliminate excessive free radicals are considered to have antioxidant activity. In this study, the DPPH radical and ABTS radical scavenging abilities were used to evaluate the in vitro antioxidant capacity of burdock roots. As shown in Figure 2A, with the increase in fermentation time, the DPPH radical scavenging ability of fermented burdock roots increased firstly and then tended to stabilize. On day 4 of fermentation, the DPPH radical scavenging activity of burdock root extract increased to 95.16%, representing a 1.69-fold enhancement compared with the unfermented control (56.46%); this difference was statistically highly significant (*p* < 0.01). No statistically significant difference was observed in the DPPH radical scavenging activity of fermented burdock root extract among days 4, 5, and 6 (*p* > 0.05). Similarly, the ABTS radical scavenging activity of fermented burdock root extract increased significantly during fermentation. Following 24 h of fermentation, the activity rose from 79.23% to 95.59%; it peaked at 98.56% on day 4 and subsequently declined slightly to 96.13% on day 6 (Figure 2B). Relative to the unfermented control, the activity remained elevated by 16.90%. Solid-state fermentation with *E. cristatum* can enhance the in vitro antioxidant capacity of burdock roots. Yi et al. observed an increase in DPPH radical and ABTS radical scavenging ability when *E. cristatum* was used for solid-state fermentation of grape skin [29]. Fermentation of buckwheat by *E cristatum* also significantly enhanced its DPPH and ABTS radical scavenging activities [14]. The enhanced in vitro antioxidant capacity of *E. cristatum* fermented products is attributed to the release and biosynthesis of antioxidant-active compounds during biotransformation [9].

**Figure 3:**
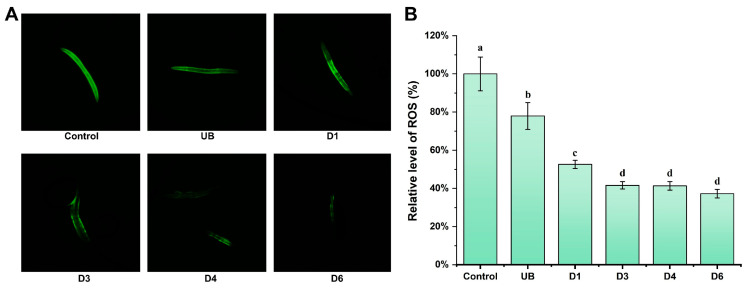
The ROS scavenging activity in living *C. elegans*. (**A**) ROS fluorescence images. (**B**) relative ROS level. UB: unfermented burdock roots; D1: burdock roots fermented for 1 day; D3: burdock roots fermented for 3 days; D4: burdock roots fermented for 4 days; D6: burdock roots fermented for 6 days. Different lowercase letters (a–d) indicate statistically significant differences (*p* < 0.05).

The ROS assay of *C. elegans* was used to evaluate the antioxidant activities in vivo of burdock root samples. In nematodes, environmental stressors and endogenous aging processes induce the generation of reactive oxygen species (ROS). Accumulation of ROS may trigger peroxidation reactions, leading to DNA damage, protein misfolding, and lipid peroxidation [30,31]. Consequently, the timely elimination of excessive ROS is instrumental in maintaining health. The ROS levels in nematodes were quantified using the HDCF-DA molecular probe. As illustrated in Figure 3A, the administration of burdock extract resulted in a reduction in ROS levels in *C. elegans*, with fermented burdock extract exhibiting a more pronounced ROS-scavenging capacity. The quantization results are shown in Figure 3B. Unfermented burdock extract eliminated 22% of ROS, while the ROS-scavenging efficacy of the fermented products enhanced progressively with the duration of fermentation. Upon supplementation with burdock extract fermented for six days, 62.7% of ROS was cleared. Aloo SO et al. reported that hempseed could mitigate ROS levels in *C. elegans*, and fermented hempseed demonstrated a superior ability to reduce ROS accumulation, indicating enhanced antioxidant activity [32]. The other research found that mulberry pomace extract decreased the ROS level in *C. elegans*, while the fermented mulberry pomace extract showed a better scavenging capacity than mulberry pomace extract [33]. Our findings are consistent with these reports, indicating that fermentation of *E. cristatum* can enhance the in vivo antioxidant capacity of burdock roots.

Notably, previous studies on burdock root fermentation have predominantly employed liquid-state fermentation with lactic acid bacteria and have primarily focused on volatile profile modulation and sensory evaluation [10,11]. In contrast, the present study utilized solid-state fermentation with *E. cristatum*—a fungus with a diverse enzyme system including cellulases, tannases, and glycosidases—and demonstrated a substantial enhancement of both in vitro and in vivo antioxidant capacity.

### 3.3. Changes in Burdock Roots Polyphenolic Compounds During the Fermentation Process

**Figure 4:**
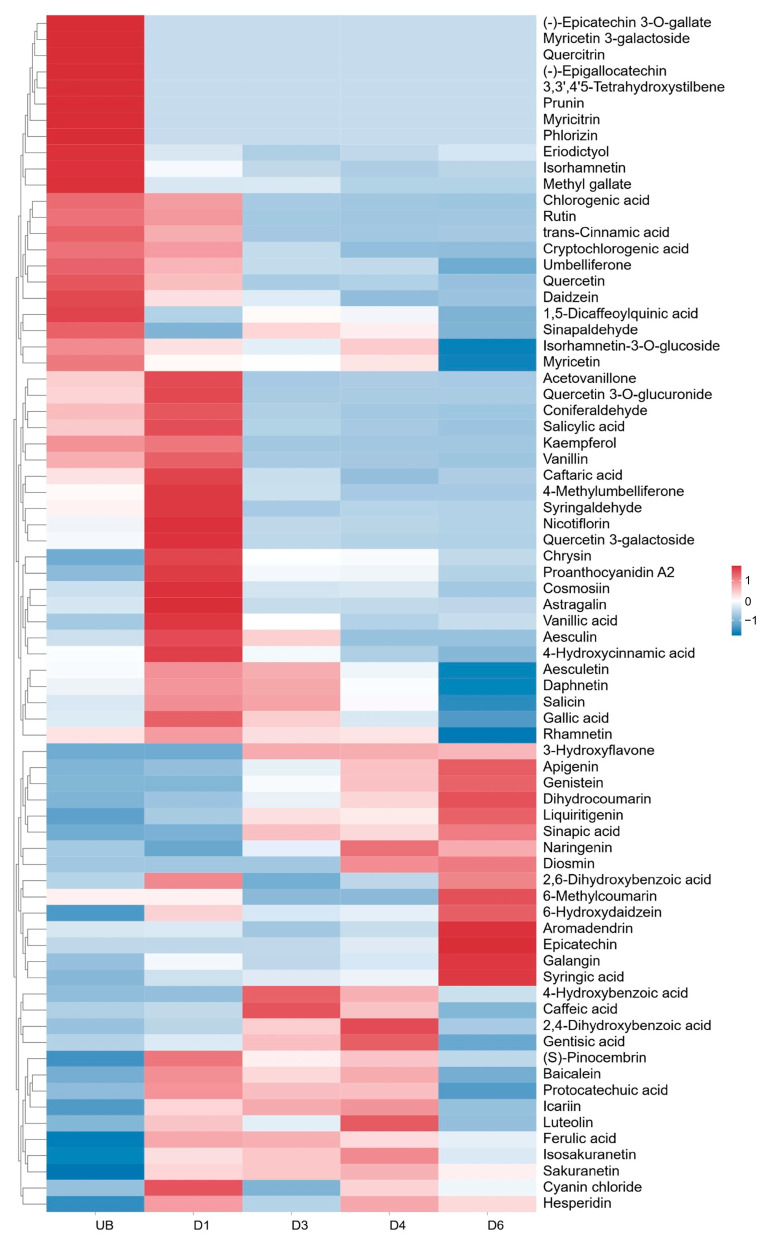
Heatmap analysis of phenolic compounds of burdock roots during fermentation. UB: unfermented burdock roots; D1: burdock roots fermented for 1 day; D3: burdock roots fermented for 3 days; D4: burdock roots fermented for 4 days; D6: burdock roots fermented for 6 days.

Previous studies have demonstrated that the abundance of phenolic compounds and their synergistic effects are essential for the antioxidant capacity of plant-based extracts [34,35]. The polyphenol extracts derived from burdock roots have been shown to exhibit potent antioxidant activity [7,36]. Researches on plant-based fermentation have demonstrated that microbial fermentation modulates the phenolic compound profile in plant-derived substrates, thereby influencing their antioxidant activity [37,38]. To assess the effects of *E. cristatum* fermentation on the phenolic profile of burdock root and to identify phenolic compounds with potential antioxidant activity, we performed quantitative and qualitative analysis of phenolic compounds using UPLC-ESI-MS/MS with external standards of authentic phenolic compounds. A total of 74 phenolic compounds were identified (Figure 4 and Table 1), predominantly flavonoids (*n* = 40) and phenolic acids (*n* = 19). Heatmap visualization in Figure 4 illustrates fermentation-induced upregulation or downregulation of these compounds based on relative abundance changes. Notably, ten flavonoids, including galangin, epicatechin, genistein, (S)-pinocembrin, 3-hydroxyflavone, liquiritigenin, sakuranetin, 6-hydroxydaidzein, diosmin, and baicalein, were exclusively detected in fermented burdock root samples, but not in the unfermented control. Among them, baicalein was present on fermentation days 1, 3, and 4, but became undetectable on day 6, whereas the other nine flavonoids persisted throughout the entire fermentation process. The majority of these newly identified compounds were aglycones, indicating that microbial enzymatic hydrolysis of flavonoid glycosides during fermentation likely released free aglycones; alternatively, certain microorganisms may have biosynthesized novel bioactive phenolic metabolites [39,40]. The concentrations of several phenolic compounds—including, but not limited to, 4-hydroxybenzoic acid, 2,4-dihydroxybenzoic acid, icariin, quercetin 3-galactoside, 4-hydroxycinnamic acid, caffeic acid, protocatechuic acid, chrysin, (S)-pinocembrin—exhibited a biphasic temporal profile during fermentation: an initial increase followed by a decline. While the peak accumulation time varied among individual compounds, most reached their maximum concentration after fermented 1 day; for example, caffeic acid peaked after fermented 3 days before declining. The early-phase elevation is likely attributable to enzymatic liberation of bound phenolics from burdock root matrix. *E. cristatum* glycosidases and cell wall-degrading enzymes release phenolic acids and flavonoid aglycones from their ester-linked or glycoside-conjugated forms [39,40]. The particularly rapid accumulation on day 1 suggests that substantial quantities of phenolics exist in bound states in unfermented burdock root and are readily accessible to microbial enzymes upon fermentation initiation. The subsequent decline suggests progressive biotransformation and metabolic consumption of these phenolics by *E. cristatum*, potentially coupled with covalent conjugation to proteins—leading to the formation of polyphenol–protein complexes that are less extractable and detectable under standard analytical conditions [41]. The variation in peak accumulation times among individual compounds, with most reaching maxima after fermented 1 day, while caffeic acid peaked after fermented 3 days, likely reflects differences in their liberation kinetics, metabolic stability, and the temporal sequence of enzyme expression during fermentation. Conversely, six compounds—3,3′,4′,5-tetrahydroxystilbene, myricetin 3-galactoside, myricitrin, phlorizin, prunin, and quercitrin—were detected exclusively in the unfermented control. Notably, these are predominantly flavonoid glycosides or glycosylated stilbenes, consistent with the glycosidase-mediated hydrolysis discussed above.

**Figure 5:**
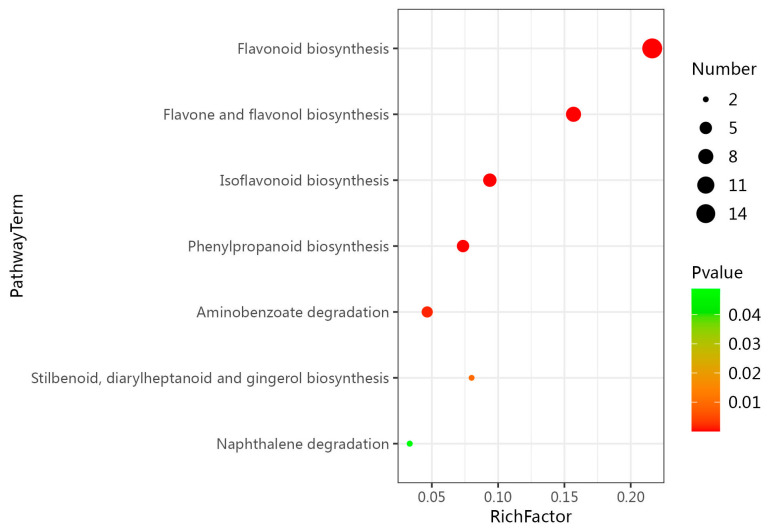
Pathway enrichment of differential metabolites between UB and D6. UB: unfermented burdock roots; D6: burdock roots fermented for 6 days.

KEGG pathway enrichment analysis of the differentially metabolites between fermented (D6) and unfermented burdock roots. Differential metabolites were selected based on the criteria of *p* < 0.05 and absolute log_2_ fold change (|log_2_(FC)|) > 1. The analysis was conducted using the KEGG database with a hypergeometric test to identify pathways significantly enriched among the differential metabolites compared with the background of all detected metabolites. Seven metabolic pathways were found to be significantly enriched at *p* < 0.05. As shown in Figure 5, these pathways are visualized in a bubble plot, where bubble size reflects the number of enriched compounds and bubble color intensity (red-to-green gradient) corresponds to higher statistical significance. Notably, six pathways were significantly enriched at *p* < 0.01 and FDR < 0.05: flavonoid biosynthesis, flavone and flavonol biosynthesis, isoflavonoid biosynthesis, phenylpropanoid biosynthesis, aminobenzoate degradation, and stilbenoid, diarylheptanoid and gingerol biosynthesis. These pathways are likely central to *E. cristatum*-mediated biotransformation of phenolic compounds in burdock roots.

### 3.4. Correlation Between Phenolic Metabolite Profiles and Antioxidant Activity in Fermented Burdock Roots

**Figure 6:**
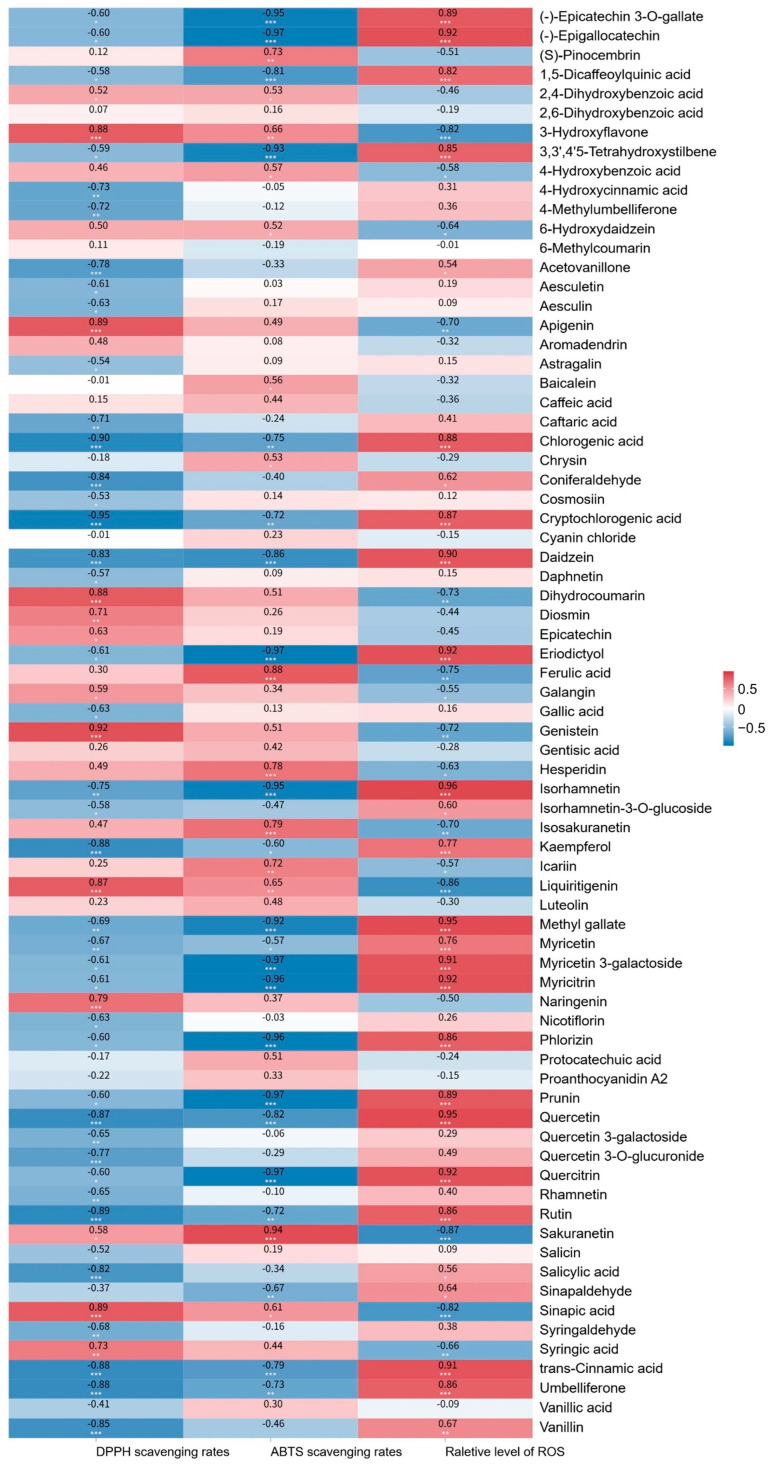
Correlation analysis between antioxidant activities and phenolic compounds of burdock roots. Asterisks indicate significance based on *p*-values (* *p* < 0.05, ** *p* < 0.01, *** *p* < 0.001).

**Figure 7:**
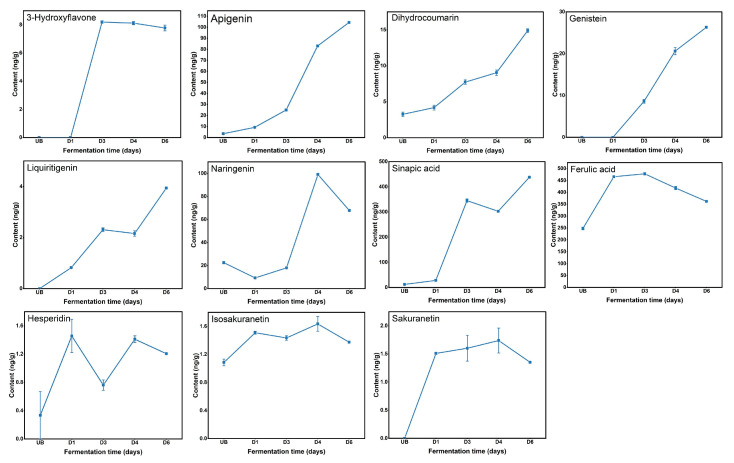
Dynamic changes in the contents of key antioxidant-associated phenolic compounds. UB: unfermented burdock roots; D1: burdock roots fermented for 1 day; D3: burdock roots fermented for 3 days; D4: burdock roots fermented for 4 days; D6: burdock roots fermented for 6 days.

To identify phenolic compounds in fermented burdock roots associated with antioxidant activity, we performed Pearson correlation analyses between the relative abundance changes of the 74 identified phenolic metabolites and three complementary antioxidant endpoints: DPPH radical scavenging activity, ABTS radical cation scavenging activity, and intracellular ROS levels in *C. elegans* (Figure 6). Correlation coefficients (r) and corresponding *p*-values were calculated. To further assess the reliability of the identified associations, the Benjamini–Hochberg false discovery rate (FDR) procedure was applied, and metabolites with FDR < 0.05 were considered significantly correlated (Table S2). Seven metabolites—sinapic acid, apigenin, naringenin, genistein, dihydrocoumarin, 3-hydroxyflavone, and liquiritigenin—exhibited significant positive correlations with DPPH scavenging capacity (*p* ≤ 0.001). Four metabolites—ferulic acid, sakuranetin, hesperidin, and isosakuranetin—showed significant positive correlations with ABTS scavenging capacity (*p* ≤ 0.001). For ROS-related activity, we evaluated associations with reduced intracellular ROS levels in *C. elegans*, as lower ROS accumulation reflects enhanced cellular antioxidant defense. Sinapic acid, 3-hydroxyflavone, liquiritigenin, and sakuranetin were significantly inversely correlated with *C. elegans* ROS levels (*p* ≤ 0.001), indicating their potential contribution to ROS scavenging efficacy. All of these significant correlations remained statistically significant after FDR correction (FDR < 0.05), confirming their reliability. Figure 7 presents the dynamic changes in the contents of phenolic compounds significantly associated with the antioxidant activity of fermented burdock roots.

Sinapic acid, 3-hydroxyflavone, liquiritigenin, and sakuranetin emerged as key modulators: all four exhibited significant positive correlations with DPPH• scavenging capacity (*p* ≤ 0.001) and *C. elegans* intracellular ROS reduction (*p* ≤ 0.001); additionally, sinapic acid, 3-hydroxyflavone, and liquiritigenin showed moderate but statistically significant positive correlations with ABTS scavenging activity (0.001 < *p* ≤ 0.05), whereas sakuranetin demonstrated a comparable association with DPPH• scavenging (0.01 < *p* ≤ 0.05). These associations all remained significant after FDR correction (FDR < 0.05), underscoring the robustness of their contribution to the overall antioxidant efficacy of fermented burdock roots. to the overall antioxidant efficacy of fermented burdock roots. Sinapic acid has been reported to attenuate ROS generation during lipogenesis and to mitigate hepatic oxidative injury through upregulation of endogenous antioxidant enzymes—including superoxide dismutase (SOD), catalase (CAT), and glutathione peroxidase (GPx) [42]. 3-Hydroxyflavone exhibits antioxidant and anti-inflammatory activities. It eliminates free radicals by inhibiting specific enzymes in the animal body [43]. Liquiritigenin, a bioactive flavanone isolated from Glycyrrhiza species, exhibits well-documented antioxidant and anti-inflammatory properties. Experimental evidence demonstrates that liquiritigenin effectively suppresses intracellular and in vivo ROS accumulation—particularly in murine models—and concurrently enhances the enzymatic activity of key endogenous antioxidants, including superoxide dismutase (SOD) and catalase (CAT). These mechanistic actions underlie its therapeutic potential in oxidative stress-associated pathologies such as heart failure and pulmonary fibrosis [44,45]. Sakuranetin is widely distributed in rice, grass trees, some herbal drugs and so on. It has antiviral, antioxidant and anti-inflammatory properties [46]. These four phenolic compounds were thus identified as promising candidate contributors to the antioxidant active components in burdock roots. Their consistent correlations across multiple assays, which remained significant after FDR correction, provide robust statistical support for their central role.

It should be noted that the correlation analysis was based on five fermentation time points. While this sampling strategy captured the critical stages of bioactivity changes and metabolite dynamics, correlation coefficients estimated from a moderate number of observations can be influenced by individual data points. Nevertheless, the associations identified here showed consistent directionality across three independent antioxidant assays and remained statistically significant after Benjamini–Hochberg FDR correction. These findings therefore provide a robust exploratory basis for prioritizing sinapic acid, 3-hydroxyflavone, liquiritigenin, and sakuranetin as candidate contributors to the antioxidant activity of fermented burdock roots. Confirmation of these associations through future studies with expanded sampling designs would further strengthen these conclusions.

### 3.5. Network Pharmacology-Based Prediction of Potential Antioxidant Mechanisms

**Figure 8:**
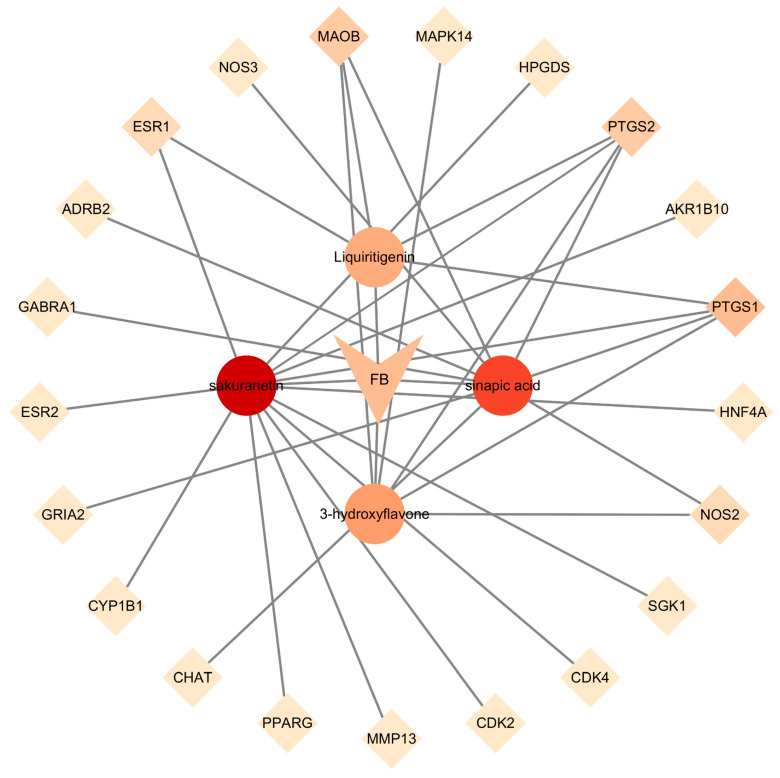
Network pharmacology analysis of antioxidant disease targets for core phenolic compounds. Node color is mapped to connectivity degree; warm colors indicate higher degree values. FB: fermented burdock roots; MAOB: Amine oxidase [flavin-containing] B; NOS3: Nitric-oxide synthase, endothelial; ESR1: Estrogen receptor alpha; ADRB2: Beta-2 adrenergic receptor; GABRA1: Gamma-aminobutyric acid receptor subunit alpha-1; ESR2: Estrogen receptor beta; GRIA2: Glutamate receptor 2; CYP1B1: Cytochrome P450 1B1; CHAT: Choline acetyltransferase; PPARG: Peroxisome proliferator-activated receptor gamma; MMP13: Matrix Metallopeptidase 13; CDK2: Cyclin-dependent kinase 2; CDK4: Cyclin-dependent kinase 4; SGK1: Serine/threonine-protein kinase 1; NOS2: Nitric oxide synthase, inducible; HNF4A: Hepatocyte nuclear factor 4-alpha; PTGS1: Prostaglandin G/H synthase 1; AKR1B10: Aldo-keto reductase family 1 member B10; PTGS2: Prostaglandin G/H synthase 2; HPGDS: Hematopoietic prostaglandin D synthase; MAPK14: Mitogen-activated protein kinase 14.

To further explore the potential mechanisms underlying the antioxidant effects of these four candidate compounds, network pharmacology analysis was subsequently performed (Figure 8). A total of 30 putative protein targets associated with the four phenolic compounds were initially retrieved from the TCMSP database. After standardization and removal of non-human or unannotated entries via UniProt, the remaining targets were screened using a Python 3.11-based keyword matching strategy for functional relevance to oxidative stress and antioxidant defense. This workflow yielded a refined set of 21 high-confidence antioxidant-related targets. Notably, all four phenolic compounds shared Prostaglandin G/H Synthase 1 (PTGS1) and Prostaglandin G/H Synthase 2 (PTGS2) as common targets.

PTGS1 and PTGS2 encode cyclooxygenase-1 (COX-1) and cyclooxygenase-2 (COX-2), respectively. These two isozymes catalyze the same key step in prostaglandin biosynthesis—the conversion of arachidonic acid to prostaglandin H_2_, a precursor for various pro-inflammatory mediators that can amplify oxidative stress. Despite this shared enzymatic function, the expression patterns and physiological roles of PTGS1 and PTGS2 are different. PTGS1 is constitutively expressed. Elevated PTGS1 expression has been consistently observed in multiple inflammatory disorders and is implicated in the dysregulation of prostaglandin-mediated inflammatory responses. In contrast, PTGS2 is typically expressed at low levels under basal conditions but is rapidly and transiently induced by inflammatory stimuli, cytokines, mitogens, and reactive oxygen species [47].

In the context of antioxidant intervention, PTGS1 may represent a particularly relevant target. As the constitutively active isoform, PTGS1 continuously governs the basal production of prostaglandins. Under conditions of chronic oxidative stress, this constitutive activity may contribute to a sustained low-grade pro-inflammatory state, thereby perpetuating oxidative damage. Consequently, inhibition of PTGS1 has the potential to provide a tonic suppression of this basal inflammatory drive [48,49]. Although PTGS2 plays a well-established role in acute inflammatory responses and can substantially amplify oxidative stress when induced, its inducible nature makes it a more prominent player in episodic inflammatory flares rather than in the chronic, low-grade oxidative stress relevant to the present study. Therefore, inhibition of PTGS1 and to a lesser extent PTGS2 may contribute to the antioxidant effects of fermented burdock root by attenuating prostaglandin-driven inflammatory cascades.

However, these findings are entirely based on computational predictions. Further in vitro and in vivo experiments are required to validate the proposed PTGS1-mediated antioxidant mechanism and to clarify the relative contributions of PTGS1 and PTGS2 to the overall bioactivity of fermented burdock root.

Among these 4 phenolic compounds, 3-hydroxyflavone, liquiritigenin and sakuranetin were new flavonoids produced after fermentation by *E. cristatum*, further verifying the mechanism by which solid-state fermentation by *E. cristatum* enhanced the antioxidant efficacy of burdock roots.

## 4. Conclusions

Solid-state fermentation using *E. cristatum* was shown to effectively upgrade the nutritional value and antioxidant properties of burdock roots. Fermentation substantially altered the nutritional composition of burdock roots, most notably yielding a 1.37-fold increase in soluble protein content. Concurrently, the antioxidant capacity was markedly enhanced, with in vitro DPPH and ABTS radical-scavenging activities both exceeding 90%, and intracellular ROS levels in vivo in *C. elegans* reduced by 62.7%. UPLC-ESI-MS/MS-based phenolic profiling revealed extensive microbial remodeling, with 10 flavonoids exclusively detected after fermentation. Correlation analysis integrating quantitative phenolic data with DPPH, ABTS, and *C. elegans* ROS-scavenging assays identified 11 phenolics as significantly associated with antioxidant efficacy. Among these, sinapic acid, 3-hydroxyflavone, liquiritigenin, and sakuranetin were the promising candidate contributors to the enhanced observed bioactivity. Network pharmacology analysis further identified PTGS1 and PTGS2 as potential antioxidant-relevant targets commonly shared by these four phenolics, with PTGS1 highlighted because of its constitutive role in maintaining basal prostaglandin levels. Critically, 3-hydroxyflavone, liquiritigenin, and sakuranetin were undetectable in unfermented roots, directly evidencing *E. cristatum*-driven biosynthesis or structural modification of antioxidant flavonoids. Future studies should focus on experimentally validating the causal contribution of the identified candidate phenolics and on confirming the predicted PTGS1/PTGS2-mediated antioxidant mechanisms in vitro and in vivo.

Based on these findings, these results suggested that fermentation by *E. cristatum* was a promising strategy to upgrade the antioxidant capacity and nutritive value of burdock roots, developing new perspectives for the microbial biotransformation of plant materials into antioxidant-rich functional ingredients.

## Figures and Tables

**Table 1:** The analysis of phenolic compositions.

Metabolites	Formula	Additive Ion	RT (min)	Contents (ng/g)				
				UB	D1	D3	**D4**	**D6**
(-)-Epicatechin 3-O-gallate	C22H18O10	[M−H]^−^	4.7	0.88 ± 0.12	nd **	nd **	nd **	nd **
(-)-Epigallocatechin	C15H14O7	[M−H]^−^	3.4	2.86 ± 0.31	nd	nd	nd	nd
(S)-Pinocembrin	C15H12O4	[M−H]^−^	13.2	nd	23.66 ± 0.58 ***	14.19 ± 2.39 **	17.67 ± 3.46 *	8.14 ± 0.49 **
1,5-Dicaffeoylquinic acid	C25H24O12	[M−H]^−^	4.1	28,040.83 ± 1824.03	13,253.77 ± 545.13 **	17,647.8 ± 663.51 **	16,597.24 ± 188.89 **	10,616.12 ± 510.74 **
2,4-Dihydroxybenzoic acid	C7H6O4	[M−H]^−^	4.2	412.53 ± 19.35	596.31 ± 16.69 ***	1304.04 ± 202.21	2089.66 ± 304.46	503.68 ± 13.87 **
2,6-Dihydroxybenzoic acid	C7H6O4	[M−H]^−^	4.97	8.61 ± 0.61	14.37 ± 0.43 ***	6.81 ± 1.45	8.73 ± 0.91	14.47 ± 0.57 ***
3-Hydroxyflavone	C15H10O3	[M−H]^+^	13.7	nd	nd	8.2 ± 0.09 ***	8.12 ± 0.12 ***	7.78 ± 0.2 ***
3,3′,4′5-Tetrahydroxystilbene	C14H12O4	[M−H]^−^	5.2	1.54 ± 0.43	nd *	nd *	nd *	nd *
4-Hydroxybenzoic acid	C7H6O3	[M−H]^−^	3.7	254.24 ± 7.08	280.14 ± 7.6 *	2091.22 ± 235.17 **	1544.81 ± 243.42 *	628.67 ± 23.37 ***
4-Hydroxycinnamic acid	C9H8O3	[M−H]^−^	4.6	2673.62 ± 67.71	7073.89 ± 290.34 ***	2525.19 ± 705.95	1072.08 ± 648.66 *	289.44 ± 3.24 ***
4-Methylumbelliferone	C10H8O3	[M−H]^+^	5.83	7.83 ± 0.2	24.64 ± 1.79 **	2.9 ± 1.48 *	nd ***	nd ***
6-Hydroxydaidzein	C15H10O5	[M−H]^−^	5.8	nd	0.85 ± 0.52	0.49 ± 0.52	0.55 ± 0.03	1.34 ± 0.09
6-Methylcoumarin	C10H8O2	[M−H]^+^	8.98	1.92 ± 0.26	1.91 ± 0.14	nd **	nd **	4.45 ± 0.32 ***
Acetovanillone	C9H10O3	[M−H]^+^	5.05	114.25 ± 0.15	195.88 ± 11.82 **	30.46 ± 8.17 **	32.84 ± 2.47 ***	31.69 ± 12.57 **
Aesculetin	C9H6O4	[M−H]^−^	4	261.84 ± 19.38	306.8 ± 26.64	296.51 ± 11.05	258.07 ± 14.96	193.93 ± 13.17
Aesculin	C15H16O9	[M−H]^+^	3.34	4.86 ± 0.7	28.8 ± 0.96 ***	15.08 ± 2.44	nd **	nd **
Apigenin	C15H10O5	[M−H]^−^	8.7	3.48 ± 0.16	9.2 ± 0.53 **	38.06 ± 22.74	70.35 ± 21.73 *	108.47 ± 7.09 **
Aromadendrin	C15H12O6	[M−H]^−^	5.9	2.03 ± 0.14	2.1 ± 0.62	1.14 ± 0.42	1.76 ± 0.81	6.53 ± 0.94 *
Astragalin	C21H20O11	[M−H]^−^	5.1	10.72 ± 2.22	124.81 ± 9 **	3.99 ± 1.98 *	1.97 ± 0.73 *	nd *
Baicalein	C15H10O5	[M−H]^−^	9.7	nd	19.57 ± 4.53 *	13.61 ± 3.88 *	17.34 ± 6 *	nd
Caffeic acid	C9H8O4	[M−H]^−^	4	3697.82 ± 194.04	4560.6 ± 213.99 **	16,809.95 ± 2295.23 **	10,982.39 ± 2325.36 *	1728.11 ± 84.32 ***
Caftaric acid	C13H12O9	[M−H]^−^	3.5	3.21 ± 0.37	4.73 ± 0.57 *	2.41 ± 0.69	1.95 ± 0.27 *	2.16 ± 0.58
Chlorogenic acid	C16H18O9	[M−H]^−^	3.6	17,567.81 ± 940.86	13,998.36 ± 749.96 **	625.68 ± 153.6 ***	136.74 ± 144.26 ***	50.74 ± 1.65 ***
Chrysin	C15H10O4	[M−H]^−^	12.9	3.25 ± 0.1	152.75 ± 4.94 ***	63.61 ± 2.17 ***	61.89 ± 8.07 **	36.96 ± 1.67 ***
Coniferaldehyde	C10H10O3	[M−H]^+^	5.77	280.4 ± 9.41	451.33 ± 13.32 ***	34.26 ± 10.61 ***	17.78 ± 5.31 ***	12.61 ± 0.18 ***
Cosmosiin	C21H20O10	[M−H]^−^	5.3	1.13 ± 0.22	8.82 ± 0.38 ***	1.33 ± 0.39	1.45 ± 0.47	nd *
Cryptochlorogenic acid	C16H18O9	[M−H]^−^	3.6	30,185.8 ± 1972.9	25,028.18 ± 1193.76 *	5657.58 ± 15.82 ***	529.24 ± 23.87 ***	184.86 ± 8.83 **
Cyanin chloride	C27H31ClO16	[M−H]^+^	2.94	0.48 ± 0.24	1.82 ± 0.57	0.37 ± 0.16	1.19 ± 0.12	0.89 ± 1.25
Daidzein	C15H10O4	[M−H]^+^	6.69	6.16 ± 1.68	3.35 ± 0.69	2.16 ± 0.87 *	0.84 ± 0.4 *	0.99 ± 0.52 *
Daphnetin	C9H6O4	[M−H]^−^	4	376.2 ± 17.06	462.98 ± 32.95 *	451.44 ± 16.58 **	385.01 ± 17.44	259.82 ± 13.05 ***
Dihydrocoumarin	C9H8O2	[M−H]^−^	4.6	3.26 ± 0.31	4.19 ± 0.33 *	6.73 ± 1.76	9.04 ± 0.38 ***	13.83 ± 1.85 **
Diosmin	C28H32O15	[M−H]^−^	5.2	nd	nd	nd	0.56 ± 0.72	0.61 ± 0.05
Epicatechin	C15H14O6	[M−H]^−^	4	nd	nd	0.19 ± 0.33	4.63 ± 2.65	40.57 ± 12.09 *
Eriodictyol	C15H12O6	[M−H]^−^	7	6.45 ± 0.25	2.43 ± 0.09 ***	1.79 ± 0.11 ***	2.06 ± 0.09 ***	2.39 ± 0.1 ***
Ferulic acid	C10H10O4	[M−H]^−^	4.9	241.22 ± 12.94	465.77 ± 2.14 ***	460.44 ± 30.97 **	425.4 ± 14.36 ***	374.98 ± 23.13 **
Galangin	C15H10O5	[M−H]^−^	13.3	nd	120.65 ± 7.74 **	50.55 ± 33.99	83.24 ± 30.58 *	413.98 ± 64.68 **
Gallic acid	C7H6O5	[M−H]^−^	2.4	967.35 ± 49.52	2218.53 ± 189.82 **	1512.73 ± 158.65 *	945.39 ± 288.3	179.13 ± 14.78 ***
Genistein	C15H10O5	[M−H]^+^	8.7	nd	nd	10.7 ± 3.63 *	17.78 ± 4.98 *	27.24 ± 1.65 **
Gentisic acid	C7H6O4	[M−H]^−^	3.8	102.59 ± 10.16	158.31 ± 9.53 **	314.25 ± 39.25 **	463.22 ± 44.17 **	nd **
Hesperidin	C28H34O15	[M−H]^−^	9.27	0.33 ± 0.33	1.46 ± 0.24 *	0.76 ± 0.07	1.41 ± 0.05 *	1.21 ± 0.01
Icariin	C33H40O15	[M−H]^−^	7.8	89.12 ± 12.41	138.44 ± 2.42 *	149.81 ± 11.13 **	155.12 ± 5.87 **	103.88 ± 18.79
Isorhamnetin	C16H12O7	[M−H]^−^	9.4	23.2 ± 0.35	8.86 ± 0.22 ***	5.4 ± 0.27 ***	4.24 ± 1.08 ***	4.98 ± 0.07 ***
Isorhamnetin-3-O-glucoside	C22H22O12	[M−H]^−^	5.2	1.51 ± 0.43	1.08 ± 0.19	0.81 ± 0.09	1.19 ± 0.37	nd *
Isosakuranetin	C16H14O5	[M−H]^−^	13	1.09 ± 0.05	1.51 ± 0.02 **	1.56 ± 0.21	1.67 ± 0.16 *	1.38 ± 0.01 **
Kaempferol	C15H10O6	[M−H]^−^	9	10.55 ± 0.37	11.93 ± 0.37	nd ***	nd ***	nd ***
Liquiritigenin	C15H12O4	[M−H]^−^	6.9	nd	0.82 ± 0.01	2.31 ± 0.07 *	2.16 ± 0.1 *	3.94 ± 0.02 **
Luteolin	C15H10O6	[M−H]^−^	7.2	2.87 ± 0.19	7.12 ± 0.44 ***	4.95 ± 2.03	9.72 ± 3.1	3.16 ± 0.22
Methyl gallate	C8H8O5	[M−H]^−^	3.8	15.62 ± 1.52	1.87 ± 0.11 **	1.96 ± 0.34 **	nd **	nd **
Myricetin	C15H10O8	[M−H]^−^	5.7	4.87 ± 0.13	2.92 ± 0.19 ***	2.85 ± 0.06 ***	3.26 ± 0.29 **	nd **
Myricetin 3-galactoside	C21H20O13	[M−H]^+^	4.27	9.82 ± 0.9	nd **	nd **	nd **	nd **
Myricitrin	C21H20O12	[M−H]^−^	4.6	311.61 ± 13.86	nd ***	nd ***	nd ***	nd ***
Naringenin	C15H12O5	[M−H]^−^	8.6	22.57 ± 0.98	9.29 ± 0.87 ***	38.35 ± 35.3	82.92 ± 28	67.77 ± 0.67 ***
Nicotiflorin	C27H30O15	[M−H]^−^	4.8	9.21 ± 1.41	40.49 ± 0.43 ***	2.5 ± 0.4 **	1.94 ± 0.9 **	1.25 ± 0.03 *
Phlorizin	C21H24O10	[M−H]^−^	5.7	5.24 ± 1.29	nd *	nd *	nd *	nd *
Protocatechuic acid	C7H6O4	[M−H]^−^	3.1	4545.04 ± 246.77 ***	10,383.15 ± 341.26 ***	9213.91 ± 128.55 ***	9183.19 ± 184.01 ***	3229.7 ± 136.12 **
Proanthocyanidin A2	C30H24O12	[M−H]^−^	4.8	4.65 ± 1.33	21.34 ± 6.21	9.88 ± 11.55	9.55 ± 0.13	6.61 ± 0.24
Prunin	C21H22O10	[M−H]^−^	5.3	2.76 ± 0.41	nd **	nd **	nd **	nd **
Quercetin	C15H10O7	[M−H]^−^	7.2	12.81 ± 0.34	7.85 ± 0.69 **	0.84 ± 0.08 ***	1.14 ± 0.11 ***	nd ***
Quercetin 3-galactoside	C21H20O12	[M−H]^−^	4.6	96.15 ± 11.4	405.92 ± 24.87 ***	17.4 ± 8.96 ***	5.58 ± 4.83 **	nd **
Quercetin 3-O-glucuronide	C21H18O13	[M−H]^−^	4.7	2.14 ± 0.63	4.52 ± 0.75 *	nd *	nd *	nd *
Quercitrin	C21H20O11	[M−H]^−^	5.2	591.67 ± 29.09	nd ***	nd ***	nd ***	nd ***
Rhamnetin	C16H12O7	[M−H]^−^	11.2	3.54 ± 0.05	4.65 ± 0.03 ***	3.63 ± 0.1	3.51 ± 0.03	nd
Rutin	C27H30O16	[M−H]^−^	4.5	275.7 ± 23.36	234.21 ± 13.15	14.37 ± 2.68 **	12.23 ± 0.8 **	13.19 ± 0.55 **
Sakuranetin	C16H14O5	[M−H]^−^	13	nd	1.51 ± 0.02 ***	1.6 ± 0.23 **	1.74 ± 0.22 **	1.35 ± 0.01 ***
Salicin	C13H18O7	[M−H]^−^	3.2	7.74 ± 0.65	16.08 ± 0.82 ***	14.94 ± 3.71	9.23 ± 1.8	nd **
Salicylic acid	C7H6O3	[M−H]^−^	6.1	297.26 ± 8.67	531.55 ± 13.37 ***	55.93 ± 8.69 ***	34.39 ± 8.25 ***	17.46 ± 0.15 ***
Sinapaldehyde	C11H12O4	[M−H]^−^	7.8	3.71 ± 0.4	nd **	2.13 ± 0.27 **	1.83 ± 0.19 **	nd **
Sinapic acid	C11H12O	[M−H]^−^	4.9	13.14 ± 1.63	26.9 ± 2.25 **	324.38 ± 36.53 **	285.89 ± 29.29 **	433.07 ± 8.72 ***
Syringaldehyde	C9H10O4	[M−H]^+^	4.89	1299.66 ± 18.34	2697.99 ± 75.53 ***	591 ± 128.81 **	693.9 ± 84.94 **	659.32 ± 23.09 ***
Syringic acid	C9H10O5	[M−H]^+^	4.16	382.8 ± 16.6	1273.66 ± 129.93 **	1495.44 ± 197.02 **	1680.16 ± 129.69 **	4730.62 ± 325.37 **
trans-Cinnamic acid	C9H8O2	[M−H]^+^	7.71	2488.65 ± 94.21	1745.85 ± 29.55 **	37.59 ± 6.07	22.02 ± 6.86	47.49 ± 3.2
Umbelliferone	C9H6O3	[M−H]^+^	4.94	25.37 ± 4.4	18.85 ± 1.66	8.23 ± 2.77 **	7.96 ± 1.79 *	2.29 ± 1.5 **
Vanillic acid	C8H8O4	[M−H]^−^	4.1	879.73 ± 86.36	1941.34 ± 88.18 ***	1194.76 ± 140.78 *	928.3 ± 195.53	995.08 ± 32.57
Vanillin	C8H8O3	[M−H]^+^	4.74	1534.87 ± 78.39	2188.52 ± 40.03 **	123.49 ± 1.61 **	84.61 ± 31.26 ***	37.86 ± 5.87 ***

Note: Data are shown as mean ± standard deviation of three sets of replicated trials. ‘nd’ represents that the compound was not detected. Asterisks indicate statistically significant differences compared to the unfermented control (UB): * *p* < 0.05, ** *p* < 0.01, *** *p* < 0.001.

## Data Availability

The raw data supporting the conclusions of this article will be made available by the authors on request.

## Supplementary Materials

The following supporting information can be downloaded at: https://www.mdpi.com/article/10.3390/antiox15070811/s1. Table S1: Method validation parameters of the targeted phenolic compounds. Table S2: Statistical results of Pearson correlation analysis between phenolic compounds and antioxidant endpoints.
