# Supplementary material for: Eurotium cristatum Solid-State Fermentation of Burdock Roots: Nutritional Changes, Enhanced Antioxidant Capacity, and Its Association with Phenolic Remodeling
Source: Antioxidants (Basel). 2026 Jun 28;15(7):811. doi: 10.3390/antiox15070811 (PMC13405727; doi:10.3390/antiox15070811)
Supplement: Supplementary file 1 [file antioxidants-15-00811-s001.zip › antioxidants-4347973-supplementary.pdf]

Table S1. Method validation parameters of the targeted phenolic compounds

Metabolites	r^2	LOQ (ng/mL)	Accuracy at LOQ (%)	LOD (ng/mL)	RSD (%)
(-)-Epicatechin 3-O-gallate	0.99957	2.137	104.2	0.647	0
(-)-Epigallocatechin	0.99873	2.403	117.2	0.728	0
(S)-Pinocembrin	0.99986	5.144	100.5	1.559	3.11
1,5-Dicaffeoylquinic acid	0.99944	2.282	111.3	0.691	3.99
2,4-Dihydroxybenzoic acid	0.99887	2.126	103.7	0.644	1.97
2,6-Dihydroxybenzoic acid	0.99827	1.967	96.0	0.596	6.30
3-Hydroxyflavone	0.99908	5.607	109.5	1.699	5.01
3,3',4'5-Tetrahydroxystilbene	0.99950	2.144	104.6	0.650	0
4-Hydroxybenzoic acid	0.99897	2.238	109.2	0.678	5.52
4-Hydroxycinnamic acid	0.99994	2.029	99.0	0.615	1.41
4-Methylumbelliferone	0.99986	1.998	97.5	0.605	13.37
6-Hydroxydaidzein	0.99841	1.923	93.8	0.583	0
6-Methylcoumarin	0.99925	2.295	112.0	0.695	10.71
Acetovanillone	0.99941	2.318	113.1	0.702	5.52
Aesculetin	0.99727	1.783	87.0	0.540	6.32
Aesculin	0.99966	2.028	98.9	0.614	15.60
Apigenin	0.99960	2.188	106.7	0.663	2.72
Aromadendrin	0.99968	2.108	102.8	0.639	11.10
Astragalin	0.99883	2.177	106.2	0.660	3.76
Baicalein	0.99730	5.912	115.5	1.792	0
Caffeic acid	0.99920	2.242	109.5	0.679	4.54
Caftaric acid	0.99906	2.349	114.6	0.712	9.38
Chlorogenic acid	0.99912	1.991	97.2	0.603	1.15
Chrysin	0.99757	2.424	118.3	0.734	1.96
Coniferaldehyde	0.99775	2.023	98.8	0.613	3.05
Cosmosiin	0.99933	2.192	106.9	0.664	16.62
Cryptochlorogenic acid	0.99941	2.021	98.6	0.612	1.04
Cyanin chloride	0.99982	0.889	108.5	0.269	8.52
Daidzein	0.99940	1.771	86.4	0.537	4.72
Daphnetin	0.99738	5.688	111.1	1.724	1.37
Dihydrocoumarin	0.99881	2.155	105.1	0.653	7.13
Diosmin	0.99972	1.957	95.5	0.593	0
Epicatechin	0.99783	1.897	92.5	0.575	5.76
Eriodictyol	0.99960	2.182	106.4	0.661	0.53
Ferulic acid	0.99783	2.219	108.3	0.672	1.53
Galangin	0.99852	0.853	104.1	0.258	6.53
Gallic acid	0.99903	2.257	110.1	0.684	6.72
Genistein	0.99962	2.124	103.6	0.644	11.93

Gentisic acid	0.99945	2.360	115.2	0.715	6.60
Hesperidin	0.99818	2.376	116.0	0.72	0
Icariin	0.99923	2.411	117.6	0.731	7.95
Isorhamnetin	0.99866	2.352	114.8	0.713	1.70
Isorhamnetin-3-O-glucoside	0.99945	1.915	93.4	0.580	10.26
Isosakuranetin	0.99971	2.204	107.5	0.668	1.01
Kaempferol	0.99894	5.037	98.4	1.526	0
Liquiritigenin	0.99934	2.228	108.7	0.675	9.69
Luteolin	0.99946	2.008	98.0	0.608	6.17
Methyl gallate	0.99988	2.163	105.6	0.655	6.47
Myricetin	0.99979	2.142	104.5	0.649	13.16
Myricetin 3-galactoside	0.99947	2.172	105.9	0.658	0
Myricitrin	0.99923	1.983	96.7	0.601	2.34
Naringenin	0.99940	2.214	108.0	0.671	9.82
Nicotiflorin	0.99958	2.098	102.3	0.636	10.99
Phlorizin	0.99994	5.214	101.8	1.58	0
Protocatechuic acid	0.99939	2.051	100.1	0.622	3.50
Proanthocyanidin A2	0.99409	2.141	104.5	0.649	3.21
Prunin	0.99829	2.295	111.9	0.695	0
Quercetin	0.99981	2.012	98.1	0.610	1.81
Quercetin 3-galactoside	0.99937	2.102	102.5	0.637	7.73
Quercetin 3-O-glucuronide	0.99930	2.010	98.1	0.609	4.94
Quercitrin	0.99955	2.020	98.5	0.612	4.97
Rhamnetin	0.99890	2.325	113.4	0.704	14.67
Rutin	0.99860	1.981	96.7	0.600	4.45
Sakuranetin	0.99986	2.156	105.2	0.653	2.77
Salicin	0.99945	2.012	98.2	0.610	3.52
Salicylic acid	0.99988	2.080	101.5	0.630	5.29
Sinapaldehyde	0.99931	2.243	109.4	0.680	12.22
Sinapic acid	0.99855	2.211	107.9	0.67	12.15
Syringaldehyde	0.99928	2.149	104.9	0.651	2.51
Syringic acid	0.99363	2.165	105.7	0.792	6.45
trans-Cinnamic acid	0.99883	2.261	110.4	0.685	2.99
Umbelliferone	0.99957	1.932	94.3	0.585	3.44
Vanillic acid	0.99828	2.019	98.6	6.12	4.54
Vanillin	0.99525	1.646	80.4	0.499	0.67

Note: LOQ, limit of quantification; LOD, limit of detection; RSD, relative standard deviation calculated from repeated injections of quality control (QC) samples. LOD was estimated as LOQ divided by 3.3.

Table.S2 Statistical results of Pearson correlation analysis between phenolic compounds and antioxidant endpoints.

EndPoint	Endpoint	*r*	*p*_Value	FDR
DPPH scavenging rates	(-)-Epicatechin 3-O-gallate	-0.603623691	0.017187933	0.035330752
ABTS scavenging rates	(-)-Epicatechin 3-O-gallate	-0.951637566	4.92127E-08	1.21391E-06
Raletive level of ROS	(-)-Epicatechin 3-O-gallate	0.891493812	8.10916E-06	6.32047E-05
DPPH scavenging rates	(-)-Epigallocatechin	-0.601832197	0.017610116	0.035866475
ABTS scavenging rates	(-)-Epigallocatechin	-0.969561151	2.53457E-09	1.87558E-07
Raletive level of ROS	(-)-Epigallocatechin	0.922791889	9.59319E-07	1.21625E-05
DPPH scavenging rates	(S)-Pinocembrin	0.117829141	0.675784702	0.729091701
ABTS scavenging rates	(S)-Pinocembrin	0.729725651	0.00201498	0.00634483
Raletive level of ROS	(S)-Pinocembrin	-0.508744272	0.052788129	0.088112516
DPPH scavenging rates	1,5-Dicaffeoylquinic acid	-0.584693129	0.022063742	0.042966235
ABTS scavenging rates	1,5-Dicaffeoylquinic acid	-0.807796363	0.000269692	0.001069137
Raletive level of ROS	1,5-Dicaffeoylquinic acid	0.815291217	0.000212308	0.000856952
DPPH scavenging rates	2,4-Dihydroxybenzoic acid	0.52195301	0.045961332	0.077888669
ABTS scavenging rates	2,4-Dihydroxybenzoic acid	0.528508509	0.042826586	0.07427736
Raletive level of ROS	2,4-Dihydroxybenzoic acid	-0.460467885	0.084120737	0.126181106
DPPH scavenging rates	2,6-Dihydroxybenzoic acid	0.068107618	0.809419742	0.835772943
ABTS scavenging rates	2,6-Dihydroxybenzoic acid	0.15541589	0.580206525	0.657172697
Raletive level of ROS	2,6-Dihydroxybenzoic acid	-0.189501557	0.498764779	0.587294802
DPPH scavenging rates	3-Hydroxyflavone	0.876866088	1.77873E-05	0.000106724
ABTS scavenging rates	3-Hydroxyflavone	0.655359668	0.007997391	0.020175236

Raletive level of ROS	3-Hydroxyflavone	-0.815390229	0.000211623	0.000856952
DPPH scavenging rates	3,3',4'5-Tetrahydroxystilbene	-0.593269472	0.019739149	0.038779566
ABTS scavenging rates	3,3',4'5-Tetrahydroxystilbene	-0.927068177	6.69386E-07	9.90692E-06
Raletive level of ROS	3,3',4'5-Tetrahydroxystilbene	0.851073046	5.73809E-05	0.000265387
DPPH scavenging rates	4-Hydroxybenzoic acid	0.462335357	0.082708154	0.124906192
ABTS scavenging rates	4-Hydroxybenzoic acid	0.565702793	0.027950012	0.051280187
Raletive level of ROS	4-Hydroxybenzoic acid	-0.577975855	0.024025792	0.045980395
DPPH scavenging rates	4-Hydroxycinnamic acid	-0.729396512	0.002029202	0.00634483
ABTS scavenging rates	4-Hydroxycinnamic acid	-0.04715332	0.867470742	0.887458547
Raletive level of ROS	4-Hydroxycinnamic acid	0.306060978	0.267246628	0.351057701
DPPH scavenging rates	4-Methylumbelliferone	-0.720607526	0.002439834	0.007319503
ABTS scavenging rates	4-Methylumbelliferone	-0.117538423	0.676544551	0.729091701
Raletive level of ROS	4-Methylumbelliferone	0.359924458	0.187591719	0.25724527
DPPH scavenging rates	6-Hydroxydaidzein	0.496042153	0.060027758	0.096566394
ABTS scavenging rates	6-Hydroxydaidzein	0.515135826	0.049397804	0.083078124
Raletive level of ROS	6-Hydroxydaidzein	-0.641073161	0.010010998	0.024156974
DPPH scavenging rates	6-Methylcoumarin	0.10910784	0.698703005	0.749333657
ABTS scavenging rates	6-Methylcoumarin	-0.193788808	0.488916417	0.5835454
Raletive level of ROS	6-Methylcoumarin	-0.007636797	0.978450714	0.982878092
DPPH scavenging rates	Acetovanillone	-0.776524174	0.000662473	0.002451149
ABTS scavenging rates	Acetovanillone	-0.326061637	0.235607433	0.315089458
Raletive level of ROS	Acetovanillone	0.540021207	0.037709092	0.066439828

DPPH scavenging rates	Aesculetin	-0.613373778	0.015024956	0.033355402
ABTS scavenging rates	Aesculetin	0.0264578	0.925429915	0.938107038
Raletive level of ROS	Aesculetin	0.187531424	0.503321265	0.588091162
DPPH scavenging rates	Aesculin	-0.625753387	0.012588584	0.028636828
ABTS scavenging rates	Aesculin	0.167321308	0.551148416	0.637265356
Raletive level of ROS	Aesculin	0.091253732	0.74636303	0.784302417
DPPH scavenging rates	Apigenin	0.893390208	7.26528E-06	6.20344E-05
ABTS scavenging rates	Apigenin	0.488748485	0.064497605	0.10154942
Raletive level of ROS	Apigenin	-0.701368883	0.003571861	0.009911915
DPPH scavenging rates	Aromadendrin	0.476468522	0.072560383	0.112646189
ABTS scavenging rates	Aromadendrin	0.077463992	0.783776614	0.813076674
Raletive level of ROS	Aromadendrin	-0.316059134	0.251124999	0.331843748
DPPH scavenging rates	Astragalin	-0.544823933	0.035714988	0.063429819
ABTS scavenging rates	Astragalin	0.090285656	0.74897348	0.784302417
Raletive level of ROS	Astragalin	0.149106274	0.595857312	0.663440384
DPPH scavenging rates	Baicalein	-0.012529736	0.964650358	0.973419906
ABTS scavenging rates	Baicalein	0.562414337	0.029079331	0.052484646
Raletive level of ROS	Baicalein	-0.321105285	0.243220174	0.323322626
DPPH scavenging rates	Caffeic acid	0.147174524	0.60068251	0.663440384
ABTS scavenging rates	Caffeic acid	0.436657935	0.103659129	0.152399515
Raletive level of ROS	Caffeic acid	-0.359827346	0.187719521	0.25724527
DPPH scavenging rates	Caftaric acid	-0.714108065	0.002784288	0.007824203

ABTS scavenging rates	Caftaric acid	-0.24089618	0.387100604	0.474786376
Raletive level of ROS	Caftaric acid	0.406786943	0.132383698	0.189607618
DPPH scavenging rates	Chlorogenic acid	-0.899985795	4.8763E-06	4.33015E-05
ABTS scavenging rates	Chlorogenic acid	-0.745389577	0.001424965	0.004721526
Raletive level of ROS	Chlorogenic acid	0.879053204	1.59183E-05	9.84662E-05
DPPH scavenging rates	Chrysin	-0.178310742	0.524900093	0.610093302
ABTS scavenging rates	Chrysin	0.532784904	0.040869003	0.071440304
Raletive level of ROS	Chrysin	-0.294487945	0.286669225	0.366310766
DPPH scavenging rates	Coniferaldehyde	-0.836204789	0.000102565	0.00046468
ABTS scavenging rates	Coniferaldehyde	-0.400880378	0.138645025	0.197302536
Raletive level of ROS	Coniferaldehyde	0.616567941	0.014364171	0.032210565
DPPH scavenging rates	Cosmosiin	-0.525248975	0.044364756	0.076348651
ABTS scavenging rates	Cosmosiin	0.144096922	0.608401704	0.668639497
Raletive level of ROS	Cosmosiin	0.118804873	0.673236557	0.729091701
DPPH scavenging rates	Cryptochlorogenic acid	-0.946268353	9.62936E-08	1.78143E-06
ABTS scavenging rates	Cryptochlorogenic acid	-0.719226863	0.002509985	0.007429554
Raletive level of ROS	Cryptochlorogenic acid	0.867543811	2.7923E-05	0.000151193
DPPH scavenging rates	Cyanin chloride	-0.005426529	0.98468677	0.98468677
ABTS scavenging rates	Cyanin chloride	0.226943679	0.415993216	0.501904859
Raletive level of ROS	Cyanin chloride	-0.149877964	0.593934112	0.663440384
DPPH scavenging rates	Daidzein	-0.833333737	0.000113991	0.00050612
ABTS scavenging rates	Daidzein	-0.861615468	3.6566E-05	0.000188783

Raletive level of ROS	Daidzein	0.902508322	4.15619E-06	3.84448E-05
DPPH scavenging rates	Daphnetin	-0.56955665	0.026669085	0.049752411
ABTS scavenging rates	Daphnetin	0.094046173	0.738847523	0.784302417
Raletive level of ROS	Daphnetin	0.147847015	0.599000975	0.663440384
DPPH scavenging rates	Dihydrocoumarin	0.884950726	1.1673E-05	8.35934E-05
ABTS scavenging rates	Dihydrocoumarin	0.506724576	0.053894091	0.089287225
Raletive level of ROS	Dihydrocoumarin	-0.725013728	0.002226391	0.006864707
DPPH scavenging rates	Diosmin	0.714612732	0.002756225	0.007824203
ABTS scavenging rates	Diosmin	0.259523028	0.350266259	0.43684893
Raletive level of ROS	Diosmin	-0.443748344	0.097539121	0.144357899
DPPH scavenging rates	Epicatechin	0.62696616	0.012367511	0.028599869
ABTS scavenging rates	Epicatechin	0.189500595	0.498766998	0.587294802
Raletive level of ROS	Epicatechin	-0.454100336	0.089066084	0.132702487
DPPH scavenging rates	Eriodictyol	-0.609251465	0.015912161	0.034975245
ABTS scavenging rates	Eriodictyol	-0.974391385	8.3404E-10	1.85157E-07
Raletive level of ROS	Eriodictyol	0.921786188	1.04093E-06	1.21625E-05
DPPH scavenging rates	Ferulic acid	0.30141193	0.274950885	0.356273225
ABTS scavenging rates	Ferulic acid	0.88143735	1.40697E-05	9.29538E-05
Raletive level of ROS	Ferulic acid	-0.747688944	0.001351566	0.004616118
DPPH scavenging rates	Galangin	0.594936924	0.019309858	0.038274898
ABTS scavenging rates	Galangin	0.341694404	0.212577357	0.289522536
Raletive level of ROS	Galangin	-0.547885901	0.034485524	0.061740212

DPPH scavenging rates	Gallic acid	-0.629312523	0.011948364	0.027921439
ABTS scavenging rates	Gallic acid	0.125264885	0.656450293	0.717891454
Raletive level of ROS	Gallic acid	0.161208062	0.565990567	0.647679927
DPPH scavenging rates	Genistein	0.92213057	1.01236E-06	1.21625E-05
ABTS scavenging rates	Genistein	0.505250724	0.0547118	0.089308968
Raletive level of ROS	Genistein	-0.724247362	0.002262397	0.006880167
DPPH scavenging rates	Gentisic acid	0.255391225	0.358262527	0.444325592
ABTS scavenging rates	Gentisic acid	0.419681563	0.119389643	0.173232031
Raletive level of ROS	Gentisic acid	-0.280673703	0.310918173	0.392180877
DPPH scavenging rates	Hesperidin	0.494633413	0.06087298	0.097221593
ABTS scavenging rates	Hesperidin	0.775258594	0.000684973	0.002492851
Raletive level of ROS	Hesperidin	-0.631170614	0.01162434	0.027453229
DPPH scavenging rates	Isorhamnetin	-0.746370037	0.001393284	0.004686502
ABTS scavenging rates	Isorhamnetin	-0.945508275	1.05304E-07	1.79827E-06
Raletive level of ROS	Isorhamnetin	0.962860748	9.08881E-09	2.52214E-07
DPPH scavenging rates	Isorhamnetin-3-O-glucoside	-0.576434819	0.024494045	0.046475881
ABTS scavenging rates	Isorhamnetin-3-O-glucoside	-0.465477716	0.080369458	0.123048412
Raletive level of ROS	Isorhamnetin-3-O-glucoside	0.604509174	0.016982157	0.035234008
DPPH scavenging rates	Isosakuranetin	0.468178788	0.078397207	0.120862361
ABTS scavenging rates	Isosakuranetin	0.785927201	0.000513413	0.001931824
Raletive level of ROS	Isosakuranetin	-0.698172245	0.003794926	0.010400909
DPPH scavenging rates	Kaempferol	-0.87899294	1.59675E-05	9.84662E-05

ABTS scavenging rates	Kaempferol	-0.597656295	0.018625137	0.037250275
Raletive level of ROS	Kaempferol	0.77045072	0.000776181	0.002779229
DPPH scavenging rates	Icariin	0.251120508	0.366632635	0.45218025
ABTS scavenging rates	Icariin	0.716137415	0.002672813	0.007706031
Raletive level of ROS	Icariin	-0.567601562	0.027313225	0.050529466
DPPH scavenging rates	Liquiritigenin	0.874683664	1.98303E-05	0.00011585
ABTS scavenging rates	Liquiritigenin	0.650344859	0.008664179	0.021371642
Raletive level of ROS	Liquiritigenin	-0.858396784	4.2117E-05	0.000209562
DPPH scavenging rates	Luteolin	0.232471707	0.404414748	0.490601498
ABTS scavenging rates	Luteolin	0.483988014	0.067542057	0.10559392
Raletive level of ROS	Luteolin	-0.300839171	0.275909162	0.356273225
DPPH scavenging rates	Methyl gallate	-0.694826666	0.004040112	0.010937863
ABTS scavenging rates	Methyl gallate	-0.923077367	9.37153E-07	1.21625E-05
Raletive level of ROS	Methyl gallate	0.950140632	5.97789E-08	1.32709E-06
DPPH scavenging rates	Myricetin	-0.672215572	0.006045978	0.015790673
ABTS scavenging rates	Myricetin	-0.572105804	0.025846582	0.04862662
Raletive level of ROS	Myricetin	0.761729381	0.000966693	0.003353215
DPPH scavenging rates	Myricetin 3-galactoside	-0.605752779	0.016696364	0.035234008
ABTS scavenging rates	Myricetin 3-galactoside	-0.969661575	2.48131E-09	1.87558E-07
Raletive level of ROS	Myricetin 3-galactoside	0.907336719	3.02343E-06	2.91827E-05
DPPH scavenging rates	Myricitrin	-0.605016853	0.016865036	0.035234008
ABTS scavenging rates	Myricitrin	-0.964404493	6.92279E-09	2.47978E-07

Raletive level of ROS	Myricitrin	0.917091293	1.50301E-06	1.58889E-05
DPPH scavenging rates	Naringenin	0.787933073	0.000485464	0.001858155
ABTS scavenging rates	Naringenin	0.3684807	0.176552345	0.246507048
Raletive level of ROS	Naringenin	-0.50062308	0.057338299	0.092913156
DPPH scavenging rates	Nicotiflorin	-0.62546565	0.012641483	0.028636828
ABTS scavenging rates	Nicotiflorin	-0.032999706	0.907058105	0.923701373
Raletive level of ROS	Nicotiflorin	0.26307682	0.343468982	0.430791605
DPPH scavenging rates	Phlorizin	-0.599503182	0.018170833	0.036672044
ABTS scavenging rates	Phlorizin	-0.963722262	7.81912E-09	2.47978E-07
Raletive level of ROS	Phlorizin	0.863323705	3.38754E-05	0.000179056
DPPH scavenging rates	Protocatechuic acid	-0.165348188	0.555920444	0.639452531
ABTS scavenging rates	Protocatechuic acid	0.50551133	0.054566556	0.089308968
Raletive level of ROS	Protocatechuic acid	-0.235763517	0.397601499	0.484986444
DPPH scavenging rates	Proanthocyanidin A2	-0.215816948	0.439810456	0.527772547
ABTS scavenging rates	Proanthocyanidin A2	0.328811724	0.231448106	0.311402906
Raletive level of ROS	Proanthocyanidin A2	-0.148583913	0.597160553	0.663440384
DPPH scavenging rates	Prunin	-0.604813466	0.016911883	0.035234008
ABTS scavenging rates	Prunin	-0.966093957	5.06742E-09	2.47978E-07
Raletive level of ROS	Prunin	0.891371817	8.16612E-06	6.32047E-05
DPPH scavenging rates	Quercetin	-0.873883803	2.06261E-05	0.00011741
ABTS scavenging rates	Quercetin	-0.824732657	0.000154658	0.000673216
Raletive level of ROS	Quercetin	0.946573459	9.28635E-08	1.78143E-06

DPPH scavenging rates	Quercetin 3-galactoside	-0.650872237	0.008592077	0.021371642
ABTS scavenging rates	Quercetin 3-galactoside	-0.059616205	0.832849762	0.855984477
Raletive level of ROS	Quercetin 3-galactoside	0.290178303	0.294110151	0.373099735
DPPH scavenging rates	Quercetin 3-O-glucuronide	-0.768449684	0.000816934	0.002878721
ABTS scavenging rates	Quercetin 3-O-glucuronide	-0.294231735	0.287108438	0.366310766
Raletive level of ROS	Quercetin 3-O-glucuronide	0.492511763	0.062162219	0.098571519
DPPH scavenging rates	Quercitrin	-0.604524165	0.01697869	0.035234008
ABTS scavenging rates	Quercitrin	-0.965066828	6.13699E-09	2.47978E-07
Raletive level of ROS	Quercitrin	0.919360779	1.26194E-06	1.40075E-05
DPPH scavenging rates	Rhamnetin	-0.645521442	0.009345847	0.022799759
ABTS scavenging rates	Rhamnetin	-0.100317996	0.722048329	0.770647735
Raletive level of ROS	Rhamnetin	0.395833666	0.144151122	0.203831523
DPPH scavenging rates	Rutin	-0.891179777	8.25647E-06	6.32047E-05
ABTS scavenging rates	Rutin	-0.718007206	0.002573289	0.007516713
Raletive level of ROS	Rutin	0.858199454	4.24788E-05	0.000209562
DPPH scavenging rates	Sakuranetin	0.581466022	0.022990466	0.044381595
ABTS scavenging rates	Sakuranetin	0.939070809	2.14212E-07	3.39679E-06
Raletive level of ROS	Sakuranetin	-0.870158848	2.46903E-05	0.000137031
DPPH scavenging rates	Salicin	-0.523044268	0.045428095	0.077577209
ABTS scavenging rates	Salicin	0.188969147	0.499994223	0.587294802
Raletive level of ROS	Salicin	0.085174484	0.762797313	0.795028186
DPPH scavenging rates	Salicylic acid	-0.817542033	0.000197182	0.000825931

ABTS scavenging rates	Salicylic acid	-0.33822078	0.217566172	0.294510306
Raletive level of ROS	Salicylic acid	0.563324896	0.02876324	0.052339665
DPPH scavenging rates	Sinapaldehyde	-0.3669961	0.178436545	0.247580706
ABTS scavenging rates	Sinapaldehyde	-0.671484717	0.006121903	0.015803052
Raletive level of ROS	Sinapaldehyde	0.636327393	0.010760777	0.025687017
DPPH scavenging rates	Sinapic acid	0.886997377	1.04407E-05	7.72611E-05
ABTS scavenging rates	Sinapic acid	0.608458505	0.016087356	0.035013657
Raletive level of ROS	Sinapic acid	-0.821858347	0.000170645	0.000728522
DPPH scavenging rates	Syringaldehyde	-0.67604744	0.005659977	0.015138733
ABTS scavenging rates	Syringaldehyde	-0.155749522	0.579383701	0.657172697
Raletive level of ROS	Syringaldehyde	0.382083472	0.159893975	0.224661155
DPPH scavenging rates	Syringic acid	0.730953062	0.001962646	0.00634483
ABTS scavenging rates	Syringic acid	0.435525686	0.104660648	0.15285963
Raletive level of ROS	Syringic acid	-0.664414682	0.006895883	0.01759639
DPPH scavenging rates	trans-Cinnamic acid	-0.881212296	1.42362E-05	9.29538E-05
ABTS scavenging rates	trans-Cinnamic acid	-0.789651372	0.00046252	0.001801393
Raletive level of ROS	trans-Cinnamic acid	0.910674502	2.40173E-06	2.42356E-05
DPPH scavenging rates	Umbelliferone	-0.883792654	1.24222E-05	8.61789E-05
ABTS scavenging rates	Umbelliferone	-0.730091441	0.001999268	0.00634483
Raletive level of ROS	Umbelliferone	0.857414835	4.39429E-05	0.000212072
DPPH scavenging rates	Vanillic acid	-0.414273385	0.124727795	0.179802405
ABTS scavenging rates	Vanillic acid	0.300766131	0.276031508	0.356273225

Raletive level of ROS	Vanillic acid	-0.09109596	0.746788292	0.784302417
DPPH scavenging rates	Vanillin	-0.853892685	5.10396E-05	0.000241081
ABTS scavenging rates	Vanillin	-0.463483503	0.081848106	0.124453969
Raletive level of ROS	Vanillin	0.67235799	0.00603127	0.015790673

Note: Pearson correlation coefficients (*r*), corresponding *p*-values, and Benjamini–Hochberg false discovery rate (FDR)-adjusted significance for correlations between individual phenolic compounds and DPPH radical scavenging activity, ABTS radical cation scavenging activity, and intracellular ROS levels in *C. elegans*.
